# Strategic alterations of posture are delayed in Parkinson’s disease patients during deep brain stimulation

**DOI:** 10.1038/s41598-021-02813-y

**Published:** 2021-12-07

**Authors:** Mitesh Patel, Maria H. Nilsson, Stig Rehncrona, Fredrik Tjernström, Måns Magnusson, Rolf Johansson, Per-Anders Fransson

**Affiliations:** 1 Faculty of Science and Engineering, University of Wolverhampton, Wolverhampton, WV1 1LY UK; 2 Department of Health Sciences, Lund University, 221 85 Lund, Sweden; 3 Memory Clinic, Skåne University Hospital, 212 24 Malmö, Sweden; 4 Clinical Memory Research Unit, Faculty of Medicine, Lund University, 221 85 Lund, Sweden; 5 Department of Neurosurgery, Lund University, 221 85 Lund, Sweden; 6 Department of Clinical Sciences, Lund University, 221 85 Lund, Sweden; 7 Department of Automatic Control, Lund University, 221 00 Lund, Sweden

**Keywords:** Diseases, Medical research, Neurology, Signs and symptoms

## Abstract

Parkinson’s disease (PD) is characterized by rigidity, akinesia, postural instability and tremor. Deep brain stimulation (DBS) of the subthalamic nucleus (STN) reduces tremor but the effects on postural instability are inconsistent. Another component of postural control is the postural strategy, traditionally referred to as the ankle or hip strategy, which is determined by the coupling between the joint motions of the body. We aimed to determine whether DBS STN and vision (eyes open vs. eyes closed) affect the postural strategy in PD in quiet stance or during balance perturbations. Linear motion was recorded from the knee, hip, shoulder and head in 10 patients with idiopathic PD with DBS STN (after withdrawal of other anti-PD medication), 25 younger adult controls and 17 older adult controls. Correlation analyses were performed on anterior–posterior linear motion data to determine the coupling between the four positions measured. All participants were asked to stand for a 30 s period of quiet stance and a 200 s period of calf vibration. The 200 s vibration period was subdivided into four 50 s periods to study adaptation between the first vibration period (30–80 s) and the last vibration period (180–230 s). Movement was recorded in patients with PD with DBS ON and DBS OFF, and all participants were investigated with eyes closed and eyes open. DBS settings were randomized and double-blindly programmed. Patients with PD had greater coupling of the body compared to old and young controls during balance perturbations (p ≤ 0.046). Controls adopted a strategy with greater flexibility, particularly using the knee as a point of pivot, whereas patients with PD adopted an ankle strategy, i.e., they used the ankle as the point of pivot. There was higher flexibility in patients with PD with DBS ON and eyes open compared to DBS OFF and eyes closed (p ≤ 0.011). During balance perturbations, controls quickly adopted a new strategy that they retained throughout the test, but patients with PD were slower to adapt. Patients with PD further increased the coupling between segmental movement during balance perturbations with DBS ON but retained a high level of coupling with DBS OFF throughout balance perturbations. The ankle strategy during balance perturbations in patients with PD was most evident with DBS OFF and eyes closed. The increased coupling with balance perturbations implies a mechanism to reduce complexity at a cost of exerting more energy. Strategic alterations of posture were altered by DBS in patients with PD and were delayed. Our findings therefore show that DBS does not fully compensate for disease-related effects on posture.

## Introduction

Parkinson’s disease (PD) is associated with motor symptoms including rigidity, akinesia, postural instability and tremor. Recent work in patients with PD has indicated a significant overlap between rest and action tremors with both variants responding well to dopaminergic treatment^1^. However, a worsening tremor is associated with poor quality of life and disability^2^. Although PD is associated with a severe depletion of dopaminergic neurons in the nigrostriatal pathway of the basal ganglia, deep brain stimulation (DBS) of the subthalamic nucleus (STN) produces significant reductions of tremor amplitude, although the exact mechanism is not known^3,4^. Several possible routes of action have been considered including the excitation and inhibition of neural circuits in and around the basal ganglia, extending to larger circuits with time^5^. An imaging study has also indicated improved thalamocortical processing from inhibition of overactive basal ganglia circuits^6^. DBS of the STN offers instantaneous improvements of tremor, reduced rigidity and bradykinesia in minutes to hours and axial symptoms, such as freezing of gait over hours or days^7^. These axial improvements involve cortical modulation and excitation of fibres to the cerebellum^8^. Although there are widespread effects from DBS in the STN, our recent studies have indicated that DBS STN does not reduce postural sway^9,10^. Given that the axial effects of DBS in the STN are varied, it would be important to consider the effect of DBS on other postural mechanisms such as the postural strategy. As the body is multi-segmented, with a number of key articulation points (i.e., knee, hip, shoulder and neck), kinematic analysis of the postural strategy may reveal such clinical alterations that are not detectable with the naked eye^11–13^. Moreover, gait and balance problems are common among people with PD as well as an increased risk of falling. Walking difficulties predict future fear of falling in people with PD^14^. Fear of falling is the strongest predictor for fall-related activity avoidance in people with PD, and it is negatively associated with health-related quality of life^15,16^. Importantly, falls are amongst the most common reasons for hospitalization among people with PD^17^.

Whole body kinematic analysis of body movement may also unravel alterations between the coupling of proximal and distal body segments in PD^18,19^. The coupling of segments identifies the phase between two regions, which can be in-phase, in counter-phase or independent. In-phase movement indicates that two body segments are moving in a coordinated fashion and in the same direction, and a counter-phase movement indicates that two segments are moving in a coordinated fashion but in opposite directions. We have previously shown that the knee and upper segments of the body are coupled in older adults from neck muscle vibration^12^. In older adults, we noted that these alterations beneficially increased flexibility in the postural system^12^.

The coordination between body segments is therefore a key indicator of strategic and impaired alterations of posture. In PD, there is often a flexed neck and trunk demonstrating kyphosis, and flexed knees in quiet standing^20^. Baston and colleagues^21^ found that patients with mild and severe PD (not treated with DBS) preferred an ankle strategy similar to controls (using the ankle as a pivot), but when they felt more unsteady, there was a shift to a hip strategy (using the hip as a pivot). However, even when balance was perturbed, there were patients with PD who did not adjust their strategic approach unlike controls, and they maintained an ankle strategy^22^. Although ankle and hip strategies serve to generalize movement in the standing posture, whole body kinematic analysis may reveal an altered behavior during initial balance perturbations.

The postural strategy may also change to repeated balance perturbations through adaptation^23^. In everyday settings, we are faced with balance challenges where adaptive mechanisms and reflexes are responsible for preventing significant postural instability^24^. These mechanisms involve the basal ganglia, which scale the correct movement. The impaired function of the basal ganglia in PD means that the adaptation of postural strategies could be altered. In line with this, efficient adaptation to perturbations is impaired in PD (non-DBS)^25,26^. Furthermore, unexpected, externally-induced, balance perturbations can generate fall-protective movements^27^ and therefore, balance perturbations may reveal strategic alterations that cannot be seen in quiet stance. One method commonly used to perturb balance through the somatosensory system is vibration of skeletal muscles or tendons, such as the calf^28^. This increases the afferent signals from the muscle spindles and creates a proprioceptive illusion that the vibrated muscle is being stretched. The tonic stretch reflexes thus induced are intended to return the vibrated muscle to its perceived original length^29^, producing an increased anterior–posterior postural sway. The postural strategy may also depend on sensory information from visual, vestibular and somatosensory signals. In PD, there is also an increased reliance on visual information^30,31^, which may be evidenced during balance perturbations.

There were multiple aims of this study: (1) To capture strategic alterations of posture in patients with PD to vibration-induced perturbations; (2) To compare the strategic alterations of posture in patients with PD with DBS ON vs. DBS OFF; (3) To compare strategic alterations of posture in patients with PD vs. controls (old and young); (4) To analyze the contribution of visual cues in patients with PD and controls (old and young) by testing participants with eyes open and eyes closed; and (5) To study adaptive changes to the strategic alterations of posture using 200 s of pseudorandom vibratory stimulation. The aims were investigated after an overnight withdrawal of anti-PD medication.

## Results

### Effects of DBS, vision and adaptation on the PD subjects

**Figure 1:**
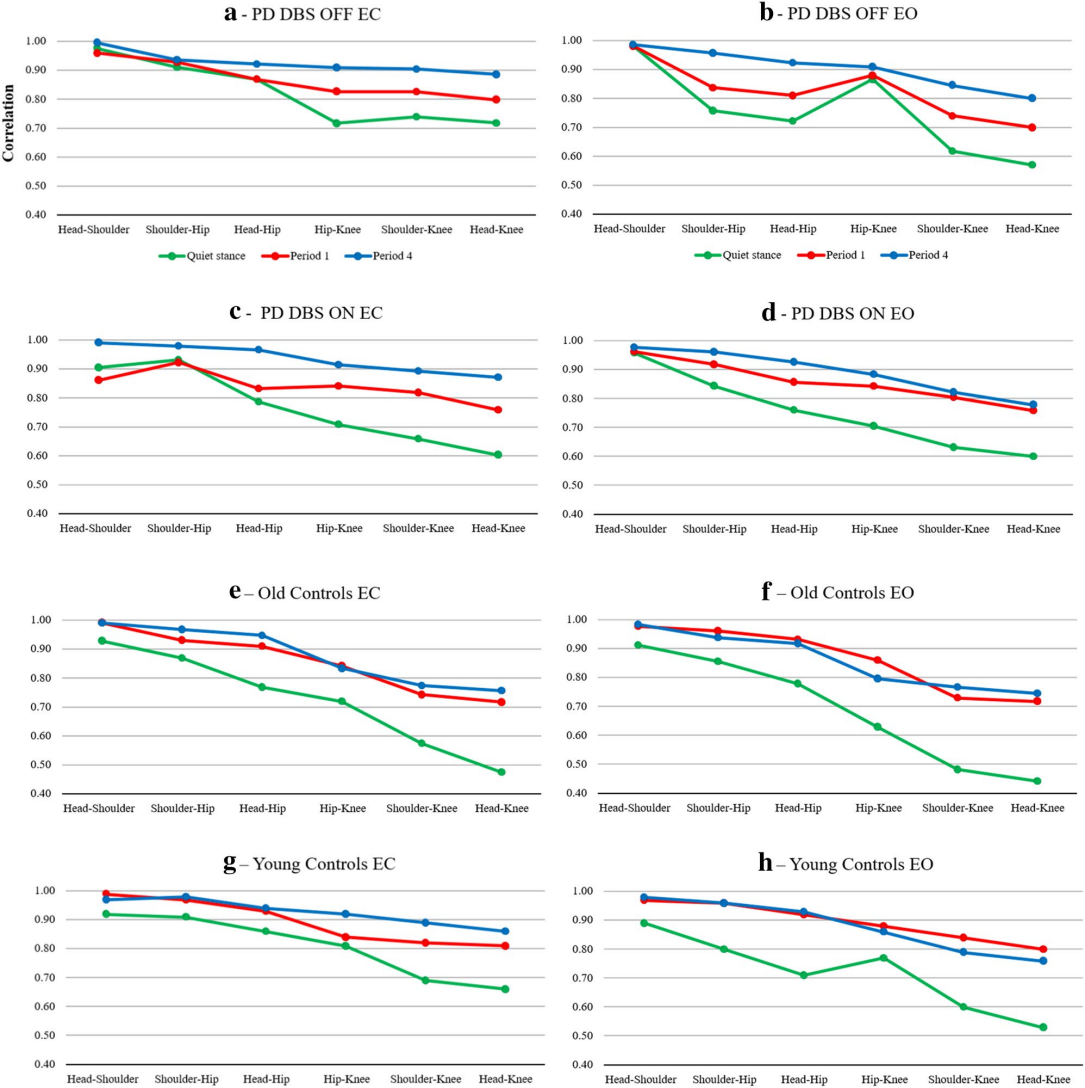
Body movement coordination (mean values) during different phases of the posturography tests for the PD subjects in DBS OFF mode (**a** eyes closed; **b** eyes open), in PD subjects in DBS ON mode (**c** eyes closed; **d** eyes open), in old controls (**e** eyes closed; **f** eyes open) and in young controls (**g** eyes closed; **h** eyes open). Note how both the older and younger controls retained the same posture in Periods 1 and Period 4 of balance perturbations, showing that they quickly adapt. The postural strategy in patients with PD took longer to finalize, mostly with DBS OFF and when standing with eyes closed.

The GLM ANOVA showed that the PD group altered their movement pattern during repeated balance perturbations (p ≤ 0.048). There was increased coupling of movement at all positions except between the shoulder–hip, see Table 1 and Fig. 1. The increased synchronicity over-time in the PD group shows a change to a more rigid ankle strategy from a multi-segmental strategy. The interaction between Vision × Adaptation (p = 0.016) for Hip–Knee shows that the synchronicity gradually increased more with eyes closed than with eyes open.

**Table 1:** DBS, vision and adaptation effects on the body movement pattern.

GLM ANOVA Statistics*^,^**	DBS	Vision	Adaptation	DBS × Vision	DBS × Adaptation	Vision × Adaptation	DBS × Vision × Adaptation
**DBS OFF vs. ON**							
Head–Shoulder	0.711 (0.2)	0.352 (1.0)	**0.048 (6.1)**	0.437 (0.7)	0.395 (0.8)	0.120 (3.3)	0.353 (1.0)
Shoulder–Hip	0.326 (1.1)	0.141 (2.9)	0.116 (3.4)	0.261 (1.5)	0.505 (0.5)	0.302 (1.3)	0.270 (1.5)
Head–Hip	0.484 (0.6)	*0.075 *(*4.6*)	**0.035 (7.3)**	0.586 (0.3)	0.773 (0.1)	0.540 (0.4)	0.357 (1.0)
Hip–Knee	0.299 (1.3)	0.787 (0.1)	**0.027 (8.5)**	0.608 (0.3)	0.406 (0.8)	**0.016 (11.0)**	0.236 (1.7)
Shoulder–Knee	0.191 (2.2)	*0.093 *(*4.0*)	**0.015 (11.3)**	0.742 (0.1)	*0.058 *(*5.5*)	0.200 (2.1)	0.322 (1.2)
Head–Knee	0.247 (1.6)	*0.056 *(*5.6*)	**0.001 (32.1)**	0.660 (0.2)	0.390 (0.9)	0.303 (1.3)	0.260 (1.5)

*Repeated measures GLM ANOVA of movement patterns with main factors “DBS”, “Vision” and “Adaptation” and their factor interactions. The F-values are presented within the parenthesis.

**Significant differences are marked with bold numbers and trends (p < 0.1) are marked with italics numbers.

### Effects of group, vision and adaptation in PD vs. old controls

GLM ANOVA showed greater coupling of the upper segments: Head–Shoulder, Shoulder–Hip and Head–Hip, in the PD group with both DBS OFF (p ≤ 0.043) and DBS ON (p ≤ 0.018) vs. old controls see Table 2 and Fig. 1.

**Table 2:** Group, vision and adaptation effect on the body movement pattern.

GLM ANOVA Statistics*^,^**	Group	Vision	Adaptation	Group × Vision	Group × Adaptation	Vision × Adaptation	Group × Vision × Adaptation
**PD OFF vs. Old**							
Head–Shoulder	0.160 (2.0)	0.199 (1.8)	**0.002 (11.7)**	0.425 (0.7)	0.102 (2.9)	**0.042 (4.7)**	**0.028 (5.5)**
Shoulder–Hip	0.251 (1.4)	0.113 (2.7)	**0.043 (4.6)**	*0.097 *(*3.0*)	*0.087 *(*3.2*)	0.278 (1.2)	**0.032 (5.3)**
Head–Hip	0.167 (2.1)	*0.066 *(*3.8*)	**0.017 (6.7)**	0.108 (2.8)	*0.061 *(*3.9*)	0.200 (1.7)	0.117 (2.7)
Hip–Knee	0.455 (0.6)	0.499 (0.5)	0.290 (1.2)	0.986 (0.0)	0.189 (1.8)	0.134 (2.4)	0.417 (0.7)
Shoulder–Knee	0.423 (0.7)	0.599 (0.3)	0.109 (2.8)	0.415 (0.7)	0.354 (0.9)	0.105 (2.9)	0.420 (0.7)
Head–Knee	0.595 (0.3)	0.360 (0.9)	*0.076 *(*3.5*)	0.246 (1.4)	0.331 (1.0)	0.116 (2.7)	0.301 (1.1)
**PD ON vs. Old**							
Head–Shoulder	*0.067 *(*3.7*)	0.659 (0.2)	**0.018 (6.4)**	0.297 (1.1)	*0.073 *(*3.5*)	*0.078 *(*3.4*)	*0.069 *(*3.6*)
Shoulder–Hip	0.979 (0.0)	0.524 (0.4)	**0.017 (6.6)**	0.445 (0.6)	*0.058 *(*4.0*)	**0.046 (44.4)**	0.172 (2.0)
Head–Hip	0.407 (0.7)	0.432 (0.6)	**0.004 (10.1)**	0.635 (0.2)	**0.020 (6.2)**	*0.071 *(*3.6*)	0.399 (0.7)
Hip–Knee	0.332 (1.0)	0.523 (0.4)	0.282 (1.2)	0.683 (0.2)	0.239 (1.5)	0.276 (1.3)	*0.071 *(*3.6*)
Shoulder–Knee	0.234 (1.5)	0.336 (1.0)	0.499 (0.5)	0.573 (0.3)	0.694 (0.2)	0.390 (0.8)	0.379 (0.8)
Head–Knee	0.324 (1.0)	0.290 (1.2)	0.205 (1.7)	0.503 (0.5)	0.684 (0.2)	0.308 (1.1)	0.357 (0.9)
**PD OFF vs. Young**							
Head–Shoulder	0.915 (0.0)	0.426 (0.7)	0.171 (2.0)	0.682 (0.2)	0.245 (1.4)	*0.075 *(*3.4*)	*0.085 *(*3.2*)
Shoulder–Hip	*0.082 *(*3.2*)	**0.002 (11.3)**	**0.010 (7.6)**	*0.052 *(*4.1*)	**0.029 (5.3)**	**0.042 (4.5)**	**0.030 (5.2)**
Head–Hip	0.130 (2.4)	**0.008 (7.9)**	**0.019 (6.1)**	0.114 (2.6)	*0.069 *(*3.6*)	0.135 (2.4)	0.198 (1.7)
Hip–Knee	0.543 (0.4)	0.747 (0.1)	**0.041 (4.6)**	0.245 (1.4)	0.525 (0.4)	**0.029 (5.2)**	0.115 (2.6)
Shoulder–Knee	0.446 (0.6)	**0.010 (7.5)**	**0.010 (7.5)**	0.919 (0.0)	**0.046 (4.3)**	0.165 (2.0)	*0.061 *(*3.8*)
Head–Knee	0.353 (0.9)	**0.004 (9.5)**	**0.024 (5.6)**	0.795 (0.1)	*0.098 *(*2.9*)	0.233 (1.5)	*0.071 *(*3.5*)
**PD ON vs. Young**							
Head–Shoulder	0.224 (1.5)	0.542 (0.4)	**0.015 (6.6)**	0.257 (1.3)	**0.045 (4.3)**	**0.035 (4.8)**	**0.038 (4.7)**
Shoulder–Hip	0.540 (0.4)	**0.008 (7.9)**	**0.001 (14.0)**	0.916 (0.0)	**0.013 (7.0)**	0.467 (0.5)	0.804 (0.1)
Head–Hip	0.544 (0.4)	0.139 (2.3)	**0.006 (8.8)**	0.962 (0.0)	**0.016 (6.4)**	0.134 (2.4)	0.217 (1.6)
Hip–Knee	0.488 (0.5)	0.228 (1.5)	**0.021 (5.9)**	0.655 (0.2)	0.463 (0.6)	0.449 (0.6)	*0.098 *(*2.9*)
Shoulder–Knee	0.399 (0.7)	**0.005 (9.3)**	**0.010 (7.5)**	0.748 (0.1)	0.358 (0.9)	0.198 (1.7)	**0.027 (5.4)**
Head–Knee	0.563 (0.3)	**0.011 (7.3)**	**0.034 (4.9)**	0.600 (0.3)	0.161 (2.1)	0.239 (1.4)	0.240 (1.4)
**Old vs. Young**							
Head–Shoulder	0.120 (2.5)	**0.034 (4.8)**	*0.084 *(*3.1*)	0.750 (0.1)	0.455 (0.6)	0.200 (1.7)	0.394 (0.7)
Shoulder–Hip	0.585 (0.3)	0.367 (0.8)	*0.092 *(*3.0*)	0.273 (1.2)	*0.054 *(*4.0*)	**0.010 (7.3)**	**0.032 (5.0)**
Head–Hip	0.689 (0.2)	0.270 (1.3)	*0.083 *(*3.2*)	0.520 (0.4)	0.121 (2.5)	0.223 (1.5)	*0.078 *(*3.3*)
Hip–Knee	*0.062 *(*3.7*)	0.731 (0.1)	0.115 (2.6)	0.177 (1.9)	0.140 (2.3)	*0.073 *(*3.4*)	0.118 (2.6)
Shoulder–Knee	**0.040 (4.5)**	0.311 (1.1)	0.169 (2.0)	0.129 (2.4)	*0.057 *(*3.8*)	0.185 (1.8)	*0.085 *(*3.1*)
Head–Knee	*0.085 *(*3.1*)	0.202 (1.7)	0.222 (1.5)	*0.091 *(*3.0*)	0.177 (1.9)	0.267 (1.3)	0.300 (1.1)

*Repeated measures GLM ANOVA of movement patterns with main factors “Group”, “Vision” and “Adaptation” and their factor interactions.

**Significant differences are marked with bold numbers and trends (p < 0.1) are marked with italics numbers.

The interaction in the PD group with DBS OFF between Vision × Adaptation (p = 0.042) for Head–Shoulder showed that the coupling gradually increased more with eyes closed compared to eyes open. The interaction between Group × Vision × Adaptation (p = 0.028) for Head–Shoulder shows reduced coupling initially in PD OFF with eyes closed compared to old controls and other test conditions. The interaction between Group × Vision × Adaptation (p = 0.032) for Shoulder–Hip shows that the coupling was initially lower in the PD group with DBS OFF with eyes open compared to old controls and other test conditions.

 The interaction between Group × Adaptation (p = 0.020) for Head–Hip in the PD group with DBS ON shows that coupling increased more compared to old controls. The interaction between Vision × Adaptation (p = 0.046) for Shoulder–Hip shows that changes in coupling were smaller with eyes closed than with eyes open in the PD group with DBS ON.

### Effects of group, vision and adaptation in PD vs. young controls

When comparing the movement patterns of the PD group vs. young controls, the GLM ANOVA revealed significant effects from vision, see Table 2 and Fig. 1. There was an increased coupling between the Shoulder–Hip, Head–Hip, Shoulder–Knee and Head–Knee with eyes closed compared to eyes open in the PD group with DBS OFF (p ≤ 0.010). There was also an increased coupling between the Shoulder–Hip, Shoulder–Knee and Head–Knee with eyes closed compared to eyes open in the PD group with DBS ON (p ≤ 0.011). Moreover, the GLM ANOVA revealed an increased coupling between all segments (the p-values for PD DBS OFF were p ≤ 0.041 and with DBS ON p ≤ 0.034), except between the Head–Shoulder in PD OFF.

The interaction in the PD group with DBS OFF between Group × Adaptation for Shoulder–Hip and Shoulder–Knee shows that the coupling increase was larger in the PD OFF group than young controls (p ≤ 0.046). The interaction in the PD group with DBS OFF between Vision × Adaptation (p = 0.042) for Shoulder–Hip shows that coupling gradually increased more with eyes open compared with eyes closed. This was not the case for Hip–Knee where the coupling increase was larger with eyes closed than with eyes open (p = 0.029). The significant interaction in the PD OFF model between Group × Vision × Adaptation (p = 0.030) for the Shoulder–Hip shows that coupling was initially lower in PD OFF with eyes open compared with young controls and other test conditions.

The interaction in PD ON between Group × Adaptation for Head–Shoulder, Shoulder–Hip and Head–Hip shows that the increase in coupling was significantly larger (p ≤ 0.045) in the PD group with DBS ON than in young controls. The interaction in the PD group with DBS ON between Vision × Adaptation (p = 0.035) for Head–Shoulder shows that the coupling gradually increased more with eyes closed compared with eyes closed. The interaction in the PD group with DBS ON between Group × Vision × Adaptation (p = 0.038) for Head–Shoulder shows that the increase in coupling was initially lower in the PD group with DBS ON with eyes closed compared to young controls and other test conditions. The interaction in the PD group with DBS ON between Group × Vision × Adaptation (p = 0.027) for Shoulder–Knee shows that coupling was initially higher in young controls with eyes closed compared with PD ON and other test conditions.

### Effects of group, vision and adaptation in old vs. young controls

When comparing the movement patterns of old vs. young controls, the GLM ANOVA revealed significant group differences. The movements of Shoulder–Knee were more coupled in young controls compared to old controls (p = 0.040), see Table 2 and Fig. 1. The movements of Head–Shoulder were more coupled with eyes closed compared with eyes open (p = 0.034).

The interaction between Vision × Adaptation (p = 0.010) for Shoulder–Hip shows that coupling gradually increased more with eyes open compared with eyes closed. The interaction between Group × Vision × Adaptation (p = 0.032) for Shoulder–Hip shows that the initial degree of coupling was lower in old controls with eyes closed compared with young controls and other test conditions.

### Effects of vision and adaptation evaluated on individual category level

#### PD with DBS OFF

The GLM ANOVA revealed that the PD group with DBS OFF made marked changes of the movement pattern during balance perturbation by significantly (p ≤ 0.023) increasing the coupling of Shoulder–Hip and Head–Knee with adaptation, see Table 3 and Fig. 1. There was no significant effect of vision.

**Table 3:** Vision and adaptation effects on body movement coordination.

GLM ANOVA statistics*^,^**	Vision	Adaptation	Vision × Adaptation
**PD OFF**			
Head–Shoulder	0.819 (0.1)	0.154 (2.7)	0.171 (2.4)
Shoulder–Hip	0.174 (2.4)	0.221 (1.9)	0.286 (1.4)
Head–Hip	0.142 (2.9)	0.100 (3.8)	0.451 (0.6)
Hip–Knee	0.551 (0.4)	*0.051 *(*5.9*)	*0.051 *(*5.9*)
Shoulder–Knee	0.222 (1.9)	**0.023 (9.2)**	0.255 (1.6)
Head–Knee	0.141 (2.9)	**0.021 (9.6)**	0.279 (1.4)
**PD ON**			
Head–Shoulder	0.576 (0.3)	0.136 (2.7)	0.189 (2.0)
Shoulder–Hip	0.174 (2.2)	**0.023 (7.5)**	0.560 (0.4)
Head–Hip	0.470 (0.6)	**0.030 (6.6)**	0.193 (2.0)
Hip–Knee	0.580 (0.3)	**0.020 (8.3)**	0.517 (0.5)
Shoulder–Knee	*0.085 *(*3.9*)	**0.012 (10.3)**	0.260 (1.5)
Head–Knee	0.227 (1.7)	**0.002 (20.2)**	0.247 (1.6)
**Old controls**			
Head–Shoulder	**0.039 (5.1)**	**0.003 (12.0)**	0.185 (1.9)
Shoulder–Hip	0.930 (0.0)	0.116 (2.8)	**0.021 (6.7)**
Head–Hip	0.790 (0.1)	*0.080 *(*3.5*)	*0.078 *(*3.6*)
Hip–Knee	0.574 (0.3)	0.445 (0.6)	0.142 (2.4)
Shoulder–Knee	0.808 (0.1)	0.461 (0.6)	0.395 (0.8)
Head–Knee	0.828 (0.0)	0.392 (0.8)	0.459 (0.6)
**Young controls**			
Head–Shoulder	0.212 (1.6)	0.337 (1.0)	0.213 (1.6)
Shoulder–Hip	**0.012 (7.4)**	0.219 (1.6)	0.463 (0.6)
Head–Hip	0.140 (2.3)	0.418 (0.7)	0.347 (0.9)
Hip–Knee	0.137 (2.4)	*0.051 *(*4.2*)	**0.034 (5.0)**
Shoulder–Knee	**0.005 (9.7)**	**0.018 (6.5)**	**0.013 (7.2)**
Head–Knee	**0.005 (9.7)**	*0.060 *(*3.9*)	0.101 (2.9)

*Repeated measures GLM ANOVA of movement patterns with main factors “Vision” and “Adaptation” and their factor interactions. The F-values are presented within the parenthesis.

**Significant differences are marked with bold numbers and trends (p < 0.1) are marked with italics numbers.

#### PD with DBS ON

The GLM ANOVA showed that the PD group with DBS ON made marked changes of the movement pattern during balance perturbation by significantly (p ≤ 0.030) increasing coupling at all locations with adaptation except between Head–Shoulder, see Table 3 and Fig. 1. There was no significant effect of vision.

#### Old controls

GLM ANOVA showed that vision significantly changed coupling in the Older Controls. Head–Shoulder was coupled more with eyes closed compared with eyes open (p = 0.039), see Table 3 and Fig. 1. Moreover, the GLM ANOVA revealed a significant increase in Head–Shoulder coupling with adaptation (p = 0.003).

The interaction between Vision × Adaptation (p = 0.021) for Shoulder–Hip shows that coupling increased more with eyes closed compared with eyes open.

#### Young controls

GLM ANOVA showed that vision affected coupling in young controls. The Shoulder–Hip, Shoulder–Knee and Head–Knee were coupled more with eyes closed compared with eyes open (p ≤ 0.012), see Table 3 and Fig. 1. There was an increase of Shoulder–Knee coupling with adaptation (p = 0.018).

The interaction between Vision × Adaptation (p ≤ 0.034) for Hip–Knee and Shoulder–Knee showed that coupling gradually increased more with eyes open compared with eyes closed.

### Effects of adaptation evaluated on individual category level

#### PD with DBS OFF

Post-hoc analyses showed that the PD group with DBS OFF made no significant changes of the movement pattern from quiet stance to balance perturbations in period 1 with eyes open or closed. Moreover, the were no changes of coupling from period 1 to period 4 with eyes open or closed, see Table 4 and Fig. 1.

**Table 4:** Movement pattern changes in different groups and conditions between quiet stance and vibration period 1 and between vibration period 1 and period 4 during tests with eyes closed and eyes open.

Body movement coordination*^,^**	Quiet stance vs. Vibration Period 1		Vibration Period 1 vs. Period 4	
	Eyes closed	Eyes open	Eyes closed	Eyes open
**PD OFF**				
Head–Shoulder	0.570 (0.98)	0.922 (1.00)	*0.031 *(*1.04*)	0.232 (1.01)
Shoulder–Hip	0.641 (1.02)	0.557 (1.11)	0.813 (1.01)	*0.049 *(*1.14*)
Head–Hip	0.945 (1.00)	*0.049 *(*1.12*)	0.469 (1.06)	0.432 (1.14)
Hip–Knee	0.313 (1.15)	0.375 (1.02)	*0.031 *(*1.10*)	0.131 (1.03)
Shoulder–Knee	0.250 (1.12)	*0.027 *(*1.20*)	*0.047 *(*1.09*)	*0.084 *(*1.14*)
Head–Knee	0.203 (1.11)	*0.084 *(*1.23*)	*0.031 *(*1.11*)	0.232 (1.15)
**PD ON**				
Head–Shoulder	0.375 (0.95)	0.492 (1.00)	**0.014 (1.15)**	*0.064 *(*1.092*)
Shoulder–Hip	0.922 (0.99)	0.432 (1.09)	**0.010 (1.06)**	**0.020 (1.05)**
Head–Hip	0.432 (1.06)	0.625 (1.13)	**0.004 (1.16)**	0.160 (1.08)
Hip–Knee	*0.084 *(*1.19*)	*0.074 *(*1.20*)	0.193 (1.09)	*0.027 *(*1.05*)
Shoulder–Knee	*0.084 *(*1.24*)	0.129 (1.27)	0.275 (1.09)	*0.098 *(*1.02*)
Head–Knee	0.193 (1.26)	0.426 (1.27)	0.131 (1.15)	*0.039 *(*1.03*)
**Old controls**				
Head–Shoulder	**< 0.001 (1.07)**	**0.017 (1.07)**	0.517 (1.00)	0.120 (1.01)
Shoulder–Hip	0.145 (1.07)	*0.034 *(*1.12*)	**0.013 (1.04)**	0.528 (0.98)
Head–Hip	*0.057 *(*1.18*)	*0.029 *(*1.20*)	*0.051 *(*1.04*)	0.821 (0.98)
Hip–Knee	**0.020 (1.17)**	**0.006 (1.37)**	0.353 (0.99)	0.821 (0.93)
Shoulder–Knee	*0.031 *(*1.29*)	*0.035 *(*1.51*)	0.190 (1.04)	0.712 (1.05)
Head–Knee	**0.004 (1.51)**	**0.017 (1.63)**	0.190 (1.05)	0.963 (1.04)
**Young controls**				
Head–Shoulder	**< 0.001 (1.08)**	**< 0.001 (1.09)**	0.560 (0.99)	0.252 (1.01)
Shoulder–Hip	**0.001 (1.06)**	**< 0.001 (1.20)**	0.353 (1.01)	0.895 (1.00)
Head–Hip	**0.002 (1.09)**	**0.001 (1.29)**	0.263 (1.01)	0.937 (1.02)
Hip–Knee	0.353 (1.04)	**0.002 (1.13)**	*0.096 *(*1.09*)	0.411 (0.98)
Shoulder–Knee	*0.075 *(*1.18*)	**< 0.001 (1.40)**	0.107 (1.08)	*0.080 *(*0.94*)
Head–Knee	**0.020 (1.23)**	**< 0.001 (1.53)**	0.220 (1.06)	0.148 (0.95)

*The quotient value between quiet stance and period 1 and between period 1 and period 4 are presented within the parenthesis. A quotient value above 1 signifies an increased synchronicity between the movements made at body sites compared.

**Bonferroni corrected significant differences are marked with bold numbers and trends (p < 0.1) are marked with italics numbers.

#### PD with DBS ON

Post-hoc analyses showed that the PD group with DBS ON made no significant changes of the movement pattern from quiet stance to period 1 with eyes open or closed, see Table 4 and Fig. 1. However, the coupling increased from period 1 to period 4 between Head–Shoulder, Shoulder–Hip and Head–Hip with eyes closed (p ≤ 0.014). Moreover, the coupling increased from period 1 to period 4 between Shoulder–Hip with eyes open (p = 0.020).

#### Old controls

Post-hoc analyses in old controls showed a significant increase in coupling from quiet stance to period 1, see Table 4 and Fig. 1. Coupling between Head–Shoulder, Hip–Knee and Head–Knee increased both with eyes closed (p ≤ 0.020) and with eyes open (p ≤ 0.017). Coupling also increased from period 1 to period 4 between Shoulder–Hip with eyes closed (p = 0.013).

#### Young controls

Post-hoc analyses in young controls showed a significant increase in coupling from quiet stance to period 1, see Table 4 and Fig. 1. Coupling increased between Head–Shoulder, Shoulder–Hip, Head–Hip and Head–Knee with eyes closed (p ≤ 0.020). Moreover, coupling increased between all segments with eyes open (p ≤ 0.002). Coupling did not change between any segments from period 1 to period 4 with eyes closed or eyes open.

## Discussion

### Strategic alterations of posture in patients with PD to vibration-induced perturbations

Postural instability is one of the cardinal signs of PD but despite its impact on patient wellbeing and implications for fall risk, biomechanical causes for such instability have not been fully identified. Our results demonstrate an altered postural strategy in patients with PD compared to both young and older controls. Patients with PD, and particularly with DBS OFF, were slower to adjust their posture to repeated balance perturbations than controls. It should be highlighted that our results refer to assessments after an overnight withdrawal of anti-PD medication (i.e., in the medication off condition).

### Strategic alterations of posture in patients with PD with DBS ON vs. DBS OFF

A major finding was a difference in response between DBS ON vs. DBS OFF to initial balance perturbations. There was no change to the postural strategy with DBS OFF, but there was an increase in coupling between segments with DBS ON from quiet stance to period 1. The increased coupling with DBS ON involved a shift to reduce flexibility, consistent with an ankle strategy. The shift to the ankle strategy with DBS ON was more evident with eyes closed compared to eyes open. As hip inflexibility in quiet stance has been attributed to postural instability in PD^21,32^ reducing flexibility of the body with DBS ON in response to the initial balance perturbations reduces the degrees of freedom of the body to control, which may simplify the corrective responses. However, small alterations of posture at one body level would produce instability at all levels. As coupling across the body was not as strong with DBS OFF eyes open compared to DBS OFF eyes closed, visual cues appear to alter the postural strategy.

The second major finding was the persistence of a flexible postural strategy in patients with PD with DBS OFF, which is consistent with others showing postural inflexibility in PD^33,34^. We have previously shown that a measure of postural control, torque variance, is the same in patients with PD with DBS ON and DBS OFF^35^, a finding that has been repeated by others^36^. It has previously been proposed that the Parkinsonian flexed posture, particularly at the knees, may compensate for posterior instability^37^.

### Strategic alterations of posture in patients with PD vs. young controls

We found that patients with PD in general had an inflexible posture compared to young controls who maintained high levels of flexibility throughout the test. The altered posture in patients with PD could be associated with postural deformity. Postural deformities such as camptocormia, Pisa syndrome and scoliosis can be found in PD^38^. Although there were no obvious signs of significant deformities in our patient cohort, we cannot rule out the effects of muscular rigidity, axial dystonia, myopathy and structural changes to the spine^39^. The flexible posture expressed by young controls enables an efficient dampening of the perturbation as each segment can be altered independently to keep the center of mass above the base of support.

### Strategic alterations of posture in patients with PD vs. older controls

There were similar responses to vibration between the patients with PD with DBS ON and old controls although in PD the alterations of posture were delayed. We found that old controls quickly adapted to balance perturbations and maintained flexibility through knee movement unlike PD patients. Previous studies have shown that proprioceptive information is down-weighted in severe PD resulting in abnormal strategies to perturbations and inappropriate strategies for the task^40,41^. Furthermore, reactions to proprioceptive stimuli are impaired in advanced stages of PD^42^. Challenges of integrating proprioceptive information cannot be ruled out and such impairments may increase reliance on other sensory cues, such as the visual and vestibular, during balance perturbations.

### The contribution of visual cues in patients with PD and controls

Patients with PD adopted a different postural strategy with eyes closed compared to eyes open. With eyes open, there was greater flexibility between the body segments, particularly with the knee, but there was an increased coupling between body segments with eyes closed. This latter finding is perhaps consistent with an increased visual contribution in PD when posture is challenged^30,42^. This altered sensory weighting in PD could be a reflection of a 'feedforward' strategy to act on possible threats to posture, which would be of higher importance given their postural instability. This alteration in sensory weighting might not generalize to early PD^43^.

### Adaptive changes to strategic alterations of posture

Patients with PD had adopted an ankle strategy by period 4 of balance perturbations with both DBS ON and DBS OFF. An ankle strategy during challenged posture is associated with co-contraction across muscles of the body. This mechanism of postural control is particularly energy demanding^44,45^ and is synonymous with fear of falling^46^ or perception of balance difficulty^47^. The finding of an ankle strategy, implying increased co-contraction in PD, is consistent with an increased antagonist muscle activity, which has been reported previously in PD^48^.

### Limitations

Our study does have limitations. The sample size of the PD group is small but is clinically well defined. A further limitation is the absence of a group of patients with PD without DBS STN. Moreover, after surgery and in real life, a combined treatment is used, i.e., reduced anti-PD medication and an ongoing STN stimulation. It would therefore be of interest to also conduct a prospective study that included assessments both with and without anti-PD medications. Furthermore, our results showed significant effects of PD vs. young and old controls. It needs to be noted that this study focuses on standing postural control using an artificial mode of perturbation rather than maintaining balance while walking and/or turning which is harder for patients with PD.

To conclude, patients with PD adopt a different postural strategy to younger and older controls. Patients with PD increase the coupling between body segments in quiet stance and further increase this coupling during balance perturbations, in line with an ankle strategy. Young and old controls maintain flexibility, which may indicate balance confidence. DBS in patients with PD altered the postural strategy but did not fully compensate for disease-effects, particularly during balance perturbations. Visual cues also altered the postural strategy but again, did not fully compensate for disease effects. Old controls quickly altered their postural strategy to the balance perturbations but patients with PD were slower.

## Methods

### Participants

Three groups were recruited: a group of PD patients; a control group of younger adults; and a control group of older adults. The study adhered to the Declaration of Helsinki and all participants provided signed, informed, consent. The study was approved by the Regional Ethical Review Board (411/2006), Lund, Sweden.

Twenty-five patients (22 men) with PD fulfilled the specific inclusion criteria of being between 59–69 years old and having been treated with bilateral STN stimulation for at least one year. From this initial group, 15 participants were excluded after declining to participate or meeting exclusion criteria: suffering from concomitant disorder decreasing postural control or causing pain, or an inability to cooperate. Thus, the final PD group were 10 adults (9 men and 1 woman) aged between 59 and 69 years (mean age 64.3 years, Standard Error of Mean (SEM) 1.3 years; mean height 1.77 m, SEM 0.02 m; and mean weight 79.6 kg, SEM 2.7 kg). None of the PD participants had camptocormia (i.e. > 45°) or PISA syndrome. The characteristics of the PD group are described in Table 5.

**Table 5:** Characteristics of patients with Parkinson’s disease (PD).

Characteristics	Median (min–max)
Age (years)	66 (59–69)
Sex	9 men, 1 woman
Disease duration (years)	18 (10–22)
Medication as l-dopa equivalent dose (mg/day) ^a^	416 (294–989)
DBS treatment duration (months)	37 (15–70)
**DBS pulse settings**	
Right	
Amplitude (V)	3.3 (2.5–4.3)
Pulse width (µs)	60 (60–90)
Frequency (Hz)	145 (100–185)
**Left**	
Amplitude (V)	3.4 (2.2–4.3)
Pulse width (µs)	60 (60–90)
Frequency (Hz)	130 (100–185)
**Positions of negative polarity contacts with reference to the intercommissural line midpoint**	
Right (mm)	
Lateral	11.7 (10.4–13.1)
Posterior	3.4 (3.0–4.0)
Inferior	2.1 (1.0–5.6)
Left (mm)	
Lateral	11.4 (9.6–13.0)
Posterior	3.5 (3.3–5.2)
Inferior	2.6 (1.2–4.2)
Intercommissural line (mm)	24.8 (23.5–25.6)
**UPDRS part III scores in anti-PD medication OFF state**^b^	
DBS OFF	
Item 20 and 21 (tremor)	2.3 (0–8.1)
Total score	41.0 (35.0–83.5)
DBS ON	
Item 20 and 21 (tremor)	0 (0–0)
Total score	21.5 (11.0–30.5)
**Berg balance scale in anti-PD medication OFF state**^b^	
DBS OFF	42 (27–50)
DBS ON	50 (41–52)
A history of falls during the past 6 months, n (%)	7 (70%)

^a^Calculated equivalent doses of Levodopa according to the method presented by Østergaard et al.^49^, and Calne^50^. All participants received L-dopa in their daily life, and 7/10 subjects received also dopamine agonists.

^b^UPDRS part III: Unified Parkinson’s disease Rating Scale, motor examination. The maximum total score is 108 points (higher scores = more severe motor symptoms). The Berg Balance scale has a scoring range from 0–56 points (higher scores = better). The evaluations were performed in anti-PD medication OFF state. All anti-Parkinsonian medications were withdrawn overnight for 10–12 h. The UPDRS assessments and balance assessments were done at the same occasion as the assessments of posture.

The control group of young adults were 25 healthy younger adults (12 men and 13 women) aged between 19 and 41 years (mean age 25.1 years, SEM 0.9 years; mean height 1.75 m, SEM 0.02 m; and mean weight 68.8 kg, SEM 2.7 kg). The control group of older adults were 17 healthy older adults (9 men and 8 women) aged between 65 and 79 years (mean 71.2 years, SEM 1.0 years; mean weight 80.1 kg, SEM 2.9 kg; and mean height 1.67 m, SEM 0.02 m). Medical assessment of individuals in the control groups confirmed the absence of vestibular dysfunction or cardiac disease, with no history of balance problems, falls or skeletal muscle atrophy. All subjects, both PD patients and control subjects, were asked to refrain from alcohol at least 48 h prior to participation in the study.

### Equipment

The movements at five anatomical bony landmarks on the right side of the subject were measured with an ultrasonic 3D-Motion Analysis system (Zebris™ CMS-HS) at 50 Hz. The “Head” marker was attached to the os zygomaticum, the “Shoulder” marker to the tuberculum majus, the “Hip” marker to the crista iliaca, the “Knee” marker to the lateral epicondyle of femur, and the “Ankle” marker to the lateral distal head of the fibula. All markers recorded its position in 3D space, i.e., anteroposterior, lateral and vertical, with a precision of about 0.4 mm.

### Procedure

Participants in the PD group were kept as in-patients the night before assessments, where anti-PD medications were withdrawn from 10 pm. The assessments were initiated about 8 o´clock in the following morning. At least 30 min prior to the first tests, the DBS was programmed to deliver STN stimulation (ON) or no STN stimulation (OFF). The DBS was programmed by a healthcare professional who was not involved in the study, to make the study double-blind. The DBS settings and the order of posturography tests with eyes closed (EC) and eyes open (EO) were randomized using a Latin Square design to avoid systematic test order biases. A counter-balanced test order design was also used to minimize systematic order effects from medication and DBS ON/OFF changes at a group level. The time to see the full effects of an altered DBS settings and withdrawal of anti-PD medications may vary. Thus, there were no tests in the first 30 min after changing the DBS settings. In both control groups, the test order of performing posturography with EC or EO first was randomized using a Latin square design.

Body movement was measured in an initial 35-s of quiet stance (quiet stance period) followed by 200 s of balance perturbations (vibration period). The randomized balance perturbations were produced by vibrators placed over the gastrocnemius muscles of both legs. The vibrators were 6 cm long and 1 cm in diameter and they produced vibration of 1.0 mm amplitude and 85 Hz frequency. The vibrations were applied ON/OFF, with durations ranging from 0.8 to 6.4 s using a pseudorandom binary sequence (PRBS)^51,52^. This randomized sequence was used to produce a sequence of unpredictable stimulation pulses of randomized duration. The stimulation sequence was identical during all tests and for all groups investigated.

In the tests, participants were asked to fold their arms across their chest and to stand in an erect and relaxed posture barefoot on a hard surface. Each participant’s heels were positioned 3 cm apart and the feet at an angle of 30° open to the front, using guidelines on the floor. Participants stood 1.5 m in front of a wall and instructed to focus on an image (6 cm × 4 cm large) placed on the wall at eye level in eyes open tests. All participants were allowed a 5-min rest between the eyes closed and eyes open tests. Participants listened to calm classical music through headphones to reduce possible movement references from external noise sources and to avoid extraneous sound distractions^53^. The participants had no prior experience of the test and they were not informed about the effects of calf vibration on their balance.

### Analysis

The relationship between the movements at different body positions were analyzed to show the postural movement pattern. Only movement in an anteroposterior direction was analyzed as calf muscle stimulation induces body movement primarily in this direction^54–56^. The movement pattern was analyzed using 6 correlation values between: head–shoulder; shoulder–hip; head–hip; hip–knee; shoulder–knee and head–knee movements, determining the synchronicity between the movements at these four locations^57,58^. The correlation values were calculated using recorded data on sample level, i.e., 1500 (30 × 50) samples for quiet stance and 2500 (50 × 50) samples for four vibration periods, which are described below. In the analysis, a correlation value of + 1.0 indicates perfect synchronicity between the two positions and in the same direction (in-phase); a correlation value of 0 indicates no relationship between the movements (movement is independent); and a correlation value of − 1 indicates perfect synchronicity between the two positions but movements are made in opposite directions (counter-phase). The correlation values were calculated using the Pearson correlation in Matlab R2019b^59^.

The movement pattern was analyzed for the quiet stance period (0–30 s) and 200 s of balance perturbations through vibration. The 200 s of balance perturbation were subdivided into four 50-s balance perturbation periods; Period 1 from 30–80 s; Period 2 from 80–130 s; Period 3 from 130–180 s and Period 4 from 180–230 s. The PRBS was designed to produce stimuli of a similar effective bandwidth in all four periods analyzed. We compared the movement pattern between the first period of balance perturbations (Period 1) and the final period of balance perturbations (Period 4) to explore changes brought about through adaptation.

### Statistical analysis

Repeated-measures GLM ANOVA was used after ensuring that all analyzed dataset combinations produced model residuals that had normal or near-normal distribution, thus validating the statistical method^60^. The main factor combinations analyzed for their effects on the movement pattern from balance perturbations were:

1. DBS (OFF vs. ON, df 1), Vision (EO vs. EC, df 1) and Adaptation (Periods 1–4, df 3).
2. Group (PD OFF vs. Old controls), Vision (EO vs. EC) and Adaptation (Periods 1–4).
3. Group (PD ON vs. Old controls), Vision (EO vs. EC) and Adaptation (Periods 1–4).
4. Group (PD OFF vs. Young controls), Vision (EO vs. EC) and Adaptation (Periods 1–4).
5. Group (PD ON vs. Young controls), Vision (EO vs. EC) and Adaptation (Periods 1–4).
6. Group (Young vs. Old controls), Vision (EO vs. EC) and Adaptation (Periods 1–4).

In analysis 1 the model parameter DBS is a Within-Subjects variable. In analyses 2–6, the model parameter Group is a Between-Subjects factor. In analyses 1–6, the model parameters Vision and Adaptation are Within-Subjects variables.

Sub-analyses using a GLM ANOVA were performed to study the effects in more detail.

1. PD with DBS OFF: Vision (EO vs. EC) and Adaptation (Periods 1–4).
2. PD with DBS ON: Vision (EO vs. EC) and Adaptation (Periods 1–4).
3. Old Controls: Vision (EO vs. EC) and Adaptation (Periods 1–4).
4. Young Controls: Vision (EO vs. EC) and Adaptation (Periods 1–4).

In analyses 1–4, the model parameters Vision and Adaptation are Within-Subjects variables.

In the post-hoc analyses, Wilcoxon Within-Subjects comparisons were used to determine whether there was a change in movement pattern from quiet stance to vibration period 1. Adaptation of the movement pattern was analyzed by comparing the movement pattern in vibration period 1 and vibration period 4. A Bonferroni correction was applied and the significant p-value level was set to p < 0.025 in post-hoc tests and at p < 0.05 in the repeated measures GLM ANOVA. Trends (p < 0.1) are also marked in the tables. Non-parametric statistics were used as not all datasets were normally distributed before or after logarithmic transformation.

## Data Availability

The datasets generated during and/or analyzed during the current study are available from the corresponding author on reasonable request.
